# Short-Term Effects of Broccoli-Derived Glucoraphanin on Recovery from Eccentric Muscle Damage: A Double-Blind Randomized Crossover Study

**DOI:** 10.3390/nu18040710

**Published:** 2026-02-23

**Authors:** Leonardo Cesanelli, Rono Thomas, Mantas Mickevičius, Audrius Sniečkus, Dalia Mickevičienė, Tomas Venckūnas, Arvydas Stasiulis, Sigitas Kamandulis

**Affiliations:** 1 Institute of Sport Science and Innovations, Lithuanian Sports University, 44225 Kaunas, Lithuania; rono.roythomas@stud.lsu.lt (R.T.); mantas.mickevicius@lsu.lt (M.M.); audrius.snieckus@lsu.lt (A.S.); dalia.mickeviciene@lsu.lt (D.M.); tomas.venckunas@lsu.lt (T.V.); 2 Department of Health Promotion and Rehabilitation, Lithuanian Sports University, 44225 Kaunas, Lithuania; arvydas.stasiulis@lsu.lt

**Keywords:** glucoraphanin, eccentric exercise, muscle damage, recovery, sulforaphane

## Abstract

**Background/Objectives**: Broccoli-derived glucoraphanin (a sulforaphane precursor that activates Nrf2 defenses) may aid repair; however, its short-term effects in humans remain unknown. This study aimed to evaluate whether short-term supplementation with broccoli-derived glucoraphanin improves recovery from exercise-induced muscle damage. We hypothesized that short-term supplementation with broccoli-derived glucoraphanin would attenuate exercise-induced muscle damage and accelerate recovery. **Methods**: In a randomized, double-blind, placebo-controlled crossover design, fifteen participants consumed either high-glucoraphanin broccoli powder (320 μg) or placebo for two weeks, followed by elbow flexor eccentric exercise. Strength, soreness, creatine kinase (CK), range of motion (ROM), arm girths, and ultrasound-assessed muscle and tendon morphology were measured at baseline, immediately post-exercise, and at 48 and 96 h post-exercise. **Results**: Significant main effects of time were observed for isometric and isokinetic torque (*p* < 0.05), CK (*p* < 0.05), soreness (*p* < 0.05), and structural swelling markers (*p* < 0.05), confirming exercise-induced muscle damage. However, there were no significant Time × Supplement interactions for any variable (*p* > 0.05), indicating that glucoraphanin did not influence recovery dynamics. **Conclusions**: These findings suggest that short-term high-dose broccoli supplementation reconstituted with hot water does not modulate recovery following eccentric muscle damage under the conditions tested, including the chosen preparation method and experimental context.

## 1. Introduction

Eccentric muscle contractions are a fundamental aspect of many physical activities, contributing significantly to the development of muscle strength, remodeling, physical fitness and performance [1]. However, these contractions also induce high mechanical stress, often resulting in exercise-induced muscle damage (EIMD) [2,3]. While EIMD is well documented in the context of athletic training, its relevance extends to the general population, particularly among untrained or sedentary individuals who may engage in daily activities involving eccentric loading, such as descending stairs, carrying groceries, or initiating resistance-based rehabilitation, which may impose substantial mechanical strain in non-adapted muscle [4,5]. The manifestations of EIMD, delayed onset muscle soreness (DOMS), strength loss, swelling, and restricted joint range of motion (ROM) can impair physical functioning and recovery [4,6,7].

Growing interest in nutritional strategies to support post-exercise recovery has spotlighted bioactive compounds with anti-inflammatory and antioxidant properties [8,9,10]. Among these, glucoraphanin, a glucosinolate found abundantly in broccoli, has gained attention for its potential to convert to sulforaphane, a potent modulator of the Nrf2 antioxidant response pathway [11,12,13]. Sulforaphane supports redox balance and regulates inflammation, making it a biologically plausible candidate for aiding muscle recovery [14,15,16,17]. While most research has focused on sulforaphane’s long-term health benefits in the context of chronic disease [10,11,17], some recent findings suggest it has a potential role in modulating acute responses to exercise-induced stress [14,15,16], potentially via rapid activation of antioxidant and cytoprotective genes during the early post-damage period.

Broccoli accumulates glucoraphanin, a stable glucosinolate that is enzymatically hydrolyzed by the plant enzyme myrosinase—or, when plant myrosinase is absent or heat-inactivated, by gut microbiota—to yield sulforaphane, a highly reactive isothiocyanate that activates the Nrf2 cytoprotective pathway and modulates redox-inflammatory signaling [11,12]. Because sulforaphane itself is chemically unstable and difficult to deliver intact, many interventions provide glucoraphanin-rich broccoli preparations, relying on endogenous or microbial myrosinase for conversion; however, conversion efficiency and downstream bioavailability are variable across individuals and influenced by processing and genetics [11,12]. In human intervention studies, broccoli-derived compounds have been delivered using a range of formulations, including whole broccoli or broccoli sprouts (fresh, cooked, or dried), lyophilized or powdered broccoli or sprout preparations, reconstituted beverages, and standardized broccoli sprout extracts, providing either sulforaphane directly or its precursor glucoraphanin, with or without active myrosinase to facilitate in vivo conversion [18].

While a two-week sulforaphane supplementation protocol in humans increased NQO1 mRNA expression in peripheral blood mononuclear cells and modestly reduced post-exercise muscle soreness and range-of-motion loss in a small parallel-group pilot study [15], no broccoli-based or sulforaphane-precursor intervention has yet tested short-term effects on integrated muscle and tendon morphology together with muscular contractile performance in a randomized double-blind crossover design study. Consequently, the acute physiological relevance of high-dose glucoraphanin intake on eccentric EIMD and recovery requires further investigation.

The present study sought to evaluate the short-term impact of broccoli powder rich in glucoraphanin on markers of EIMD and recovery following an eccentric exercise protocol targeting the elbow flexors. Using a randomized, double-blind, placebo-controlled crossover design, we assessed multiple indicators of post-exercise recovery. By incorporating both muscular and tendinous outcomes, this study aimed to provide a comprehensive picture of the extent of exercise-induced changes and the time course of recovery under supplementation with broccoli-derived glucoraphanin. We hypothesized that acute glucoraphanin supplementation would attenuate the effects of EIMD and improve recovery, with the strongest effects anticipated for muscle soreness, strength loss, and ultrasound-derived indices of muscle swelling during the 48–96 h recovery period.

## 2. Materials and Methods

### 2.1. Subjects

The present study recruited 17 healthy male volunteers aged 18–35 years who had no resistance training experience in the past 6 months, were not engaged in any structured training program for at least 12 months prior to the study, had no arm injuries in the past 2 years, no illnesses in the month prior to data collection, no self-reported chronic metabolic, cardiovascular, or musculoskeletal diseases, and no known allergies to broccoli or other cruciferous vegetables. Two volunteers were excluded because their regular work duties prevented complete participation, leaving 15 participants (mean ± standard deviation (SD)): age 26 ± 6 years; body mass 83.8 ± 13.4 kg) for analysis. Because no effect-size data from comparable crossover trials were available (only a parallel-group pilot study [15]), we recruited to a feasibility target of at least ~15 completers and used a within-subject crossover design to limit inter-individual EIMD variability, with post hoc sensitivity analysis (α = 0.05, 1−β = 0.80) showing we could detect medium Time × Supplement effects (~partial η^2^ ≥ 0.06). Participants were instructed to maintain their regular diet, abstain from exercise for one week before the experiment, and not engage in any additional physical activities during the experimental period. Supplement adherence was quantified via daily logs and confirmed by sachet count, with compliance exceeding 95% for all participants. No cases of nausea, heartburn, or other gastrointestinal discomfort were reported. Participants were surveyed retrospectively about the consumption of various cruciferous vegetables (cabbage, turnips, mustard greens, broccoli, horseradish, arugula, radishes) over the previous month, indicating frequency and approximate amounts, which indicated similar low habitual intake across participants, with no meaningful changes during the study period. Each participant read and signed a written informed consent form. The study was approved (22 May 2024) by the local Ethics Committee (Lithuanian Sports University, MNLKIN(M)-2024-574) and conducted in accordance with the principles outlined in the Declaration of Helsinki. Under Lithuanian regulations, registration of a study of this nature (nutritional supplementation without medicinal classification or drug supply) is not legally required, as it does not fall under the category of a regulated clinical drug trial [19]. Nevertheless, in the interest of transparency and adherence to best research practices, we sought to register the study at ClinicalTrials.gov. The study was registered prospectively at ClinicalTrials.gov (Identifier: NCT07373106) within the system framework as an extension of the existing protocol submission, maintaining the same methodology, outcomes, and study characteristics.

### 2.2. Study Design

Participants underwent two separate intervention periods of muscle-damaging exercise targeting the elbow flexors in a randomized crossover design: (1) following seven days of broccoli powder consumption (condition X), and (2) following an equivalent placebo regimen (condition Y). The broccoli supplementation consisted of 10 g of broccoli powder [a freeze-dried glucoraphanin-rich broccoli powder (99.5% broccoli and 0.5% mustard seed)] dissolved in 125 mL of boiling water, providing 320 μg of glucoraphanin per serving as confirmed by manufacturer (The Smarter Food Company, Norwich, UK). The broccoli powder was prepared as a hot beverage to reflect a common dietary mode of consumption and to facilitate standardized intake with meals, consistent with how such products are typically used. It was consumed while still hot or warm once daily at any time during the day for six consecutive days before the eccentric exercise, 3 h before the exercise on the testing day, and once daily at any time during the day for four days following the eccentric exercise protocol. Because the supplement was prepared using hot water, sulforaphane formation from glucoraphanin was expected to depend primarily on post-ingestion conversion. The placebo powder consisted of spinach, nettle, dill, parsley, carrot, onion, and basil, was matched in appearance and preparation, and contained no broccoli-derived bioactives. The 7-day, ~320 μg/day glucoraphanin dose (manufacturer’s standard serving) was selected for feasibility and is slightly above the median dose reported in previous studies [18]. Because hot-water reconstitution may reduce myrosinase activity, sulforaphane yield likely depended on residual enzyme activity and gut microbiota conversion, which was not measured and is acknowledged as a limitation.

Intervention order (i.e., supplement or control) was randomized in a double-blind, crossover design with a 14-day washout period, during which participants switched to the alternate supplement and repeated the same protocol using the contralateral arm. Simple randomization was performed using a random number generator for the interventions, performed by a researcher not involved in the experiment, with all participants starting the first intervention with their dominant arm to ensure equal dominant arm use between the broccoli powder and placebo conditions. Both participants and investigators conducting the assessments were blinded to supplement allocation. Supplements were provided in identical sealed sachets.

**Figure 1:**
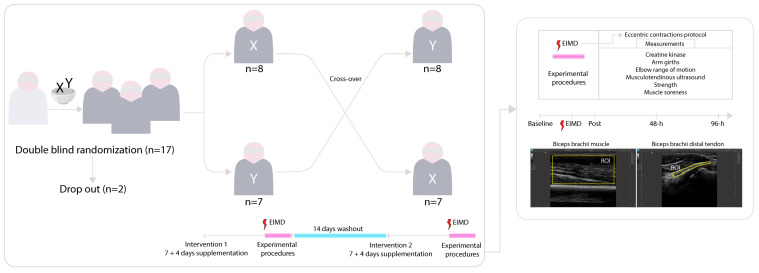
Overview of the study design. Following two dropouts, fifteen participants were double-blindly randomized to begin with either the broccoli extract (X) or the control (Y) for 7 days prior to the muscle-damaging protocol and continued consumption for 4 days thereafter. On day 7, baseline assessments were conducted, including creatine kinase levels, arm girths, elbow ROM, musculotendinous ultrasound, and strength tests. Participants then completed an eccentric exercise protocol to induce muscle damage, immediately post-exercise assessments, and subsequent follow-ups at 48 and 96 h. After a 14-day washout period, participants crossed over to the alternate condition, and the entire protocol was repeated immediately post-exercise, and at 48 and 96 h post-exercise.

Markers of muscle damage include reductions in isometric and isokinetic peak torque, restricted elbow range of motion (ROM), increased arm girth, soreness, and elevated plasma creatine kinase (CK) activity and serve as dependent variables. On the test day, a fingertip blood sample was collected for CK analysis, followed by assessments of elbow ROM, arm girth, biceps muscle soreness, and muscle strength using an isokinetic dynamometer (Biodex Medical Systems, System 4, Inc., Shirley, NY, USA). Prior to testing, participants completed a standardized warm-up on an arm ergometer (6 min, workload ≈ body mass in watts). All assessments were repeated immediately post-exercise, and at 48 and 96 h post-exercise. An overview of the experimental timeline is provided in Figure 1.

### 2.3. Muscle-Damaging Exercise

The subjects were seated on a Biodex isokinetic dynamometer with the backrest fixed at an angle of 90°, and the trunk, pelvis, and upper arm stabilized with Velcro straps. The axis of rotation was aligned with the elbow joint, and the lever arm pad was attached at the wrist. As part of the warm-up, participants completed a brief low-load simulation of the eccentric protocol prior to each trial to familiarize them with the movement and contraction mode. Subjects performed six sets of ten maximal voluntary eccentric contractions of the elbow flexors with an angular velocity of 60°/s, starting from 120° elbow flexion to full extension (the full elbow extension angle was considered as 0°), with a 1 min rest between sets. Each subject used the Biodex dynamometer to induce muscle damage in a randomly selected arm, either dominant or non-dominant. After 14 days, the same exercise was repeated with the alternative intervention on the other arm. Verbal encouragement was provided to maintain maximal effort throughout each stretch. Total eccentric work was monitored during each session to ensure protocol consistency and was comparable between the two crossover conditions; these data were not included in the Results to avoid redundancy.

### 2.4. Strength Assessment

To evaluate voluntary muscle strength, isometric (PT_ISOM_) and concentric isokinetic peak torque (PT_ISOK_) of the elbow flexors were measured using the same dynamometer (Biodex System 4) and the same setup as during the muscle-damaging exercise load. The elbow was positioned and fixed at 90° of flexion, with full elbow extension defined as 0°. Before baseline testing, participants performed several submaximal and brief maximal contractions as part of the standardized warm-up to familiarize them with the testing procedures. PT_ISOM_ was assessed first through two maximal voluntary contractions, each lasting approximately 1–2 s, with a 1 min rest between trials. After a 1 min rest, PT_ISOK_ was measured at an angular velocity of 60°/s through a 120° ROM (0°–120°), using two continuous contractions in the same setup. The highest torque value from the two trials in each condition was recorded for analysis. Verbal encouragement and real-time visual torque feedback were provided to promote maximal effort. A decline in voluntary contraction force and prolonged recovery are considered indirect markers of EIMD [20,21].

### 2.5. Range of Motion and Arm Girths

Arm circumference and elbow joint ROM were assessed according to standardized protocols. Arm girths were measured using a flexible, non-elastic 1-meter tape to the nearest 0.1 cm, with the participant standing upright. Measurements were taken at the midpoint between the acromion and olecranon processes, first with the arm relaxed and then in a fully flexed, contracted state, following the recommendations of the American College of Sports Medicine [22]. Elbow ROM was assessed using a digital goniometer (Jamar Plus+ Digital Goniometer, Performance Health, Warrenville, IL, USA) first with the arm in a neutral resting position, defined as the natural, relaxed elbow position adopted by each participant while standing, without voluntary muscle contraction [23]. Then, participants actively moved their elbow into full flexion and extension, sustaining each position for approximately five seconds while the angle was recorded.

### 2.6. Musculoskeletal Ultrasound

Biceps brachii muscle thickness (MT), cross-sectional area (CSA), and distal tendon thickness (TT) were measured using B-mode grayscale ultrasound (10–15 MHz linear-array probe, Echoblaster 128, Telemed, Vilnius, Lithuania). Imaging procedures followed the guidelines of the European Society of Musculoskeletal Radiology and prior protocols [24,25]. Participants sat with the arm supported on an armrest and a water-based transmission gel was applied to minimize transducer pressure. Transducer pressure was standardized by applying the minimum force required to obtain a clear image without hypoechogenic regions, indicating full probe aligned with the target tissue. MT and CSA were acquired at two-thirds of the distance between the acromion and antecubital crease. Three images per muscle were captured and analyzed in ImageJ (v.1.46; NIH, USA). Measurement sites were marked relative to anatomical landmarks and recorded for each participant to ensure identical probe placement and orientation across all time points. The same probe angle and limb positioning were replicated during each session. For TT, the transducer was aligned with the long axis of the distal biceps tendon using an oblique ulnar approach at the elbow joint, positioned medially to the radial tuberosity to optimize visualization of the tendon insertion. To reduce anisotropy artifacts, participants slightly bent the arm in full supination, and TT was calculated as the average of measurements taken at 5, 10, and 15 mm proximal to the radial tuberosity [25,26]. Echo intensity (EI) was analyzed from the same ultrasound images using grayscale histogram analysis in ImageJ, with values ranging from 0 (black) to 255 (white), following similar previous muscle and tendon approaches [27,28]. Specifically, a region of interest was manually selected to encompass the largest possible portion of the muscle, excluding the superficial and deep aponeuroses, and for the tendon, the central region bounded by hyperechoic borders. Both assessments were performed using longitudinal views to ensure consistent anatomical alignment across time points. All ultrasound measures were performed by a single experienced operator blinded to supplement condition throughout the study. Intrarater reliability was assessed prior to data collection on four separate occasions in two male participants; although the sample size was limited, the measurements demonstrated excellent consistency (MT: ICC = 0.97, CV = 3.46%; TT: ICC = 0.98, CV = 2.73%), supporting the reliability of the ultrasound assessments.

### 2.7. Muscle Soreness

Muscle soreness was assessed subjectively using a 10-point visual analogue scale during active movement. Each point on the scale had a written description of soreness: 0 (none), 1 (very slight), 2 (slight), 3 (mild), 4 (less than moderate), 5 (moderate), 6 (more than moderate), 7 (intense), 8 (very intense), 9 (barely tolerable), and 10 (intolerably intense). Subjects were required to evaluate the severity of soreness in their biceps brachii during 2–3 full-range eccentric–concentric contractions. Muscle soreness is widely accepted as an indirect indicator of EIMD [20,21].

### 2.8. Plasma CK Activity

Approximately 0.25 mL of capillary blood was drawn from the finger and immediately centrifuged. The plasma was then used to measure CK activity using a Spotchem™ biochemical analyzer (EZ SP-4430, Menarini Diagnostics, Winnersh-Wokingham, UK) with soft reagent strips (Arkray Factory, Inc., Shiga, Japan) reported as micro-Katalytic units per L (µkat·L^−1^). According to the analyzer manual, the normal reference range for human plasma CK using this method is 0.9–4.1 µkat·L^−1^. Elevated plasma CK activity is considered an indirect biochemical marker of muscle membrane damage [20,29].

### 2.9. Statistical Analysis

Data were analyzed using SPSS (version 30.0.0; IBM Corp., Armonk, NY, USA) and are presented as mean ± SD. Data distribution was assessed using the Shapiro–Wilk test, which confirmed normality for all variables. A repeated-measures design was used to assess the effects of eccentric EIMD and supplementation on multiple physiological outcomes. The factor ‘supplement’ represents the within-subject crossover condition, with each participant completing both supplement and placebo trials in randomized order. For all dependent variables except CK and muscle soreness, a three-way repeated-measures ANOVA was conducted with Time (Pre, Post, 48 h, 96 h), Supplement (X vs. Y), and Arm Condition (eccentrically exercised [E] vs. control [C]) as within-subject factors. For CK and muscle soreness, which were only measured in the exercised arm, a two-way repeated-measures ANOVA (Time × Supplement) was used. Additionally, as muscle soreness is ordinal, supplementary non-parametric Friedman test was also conducted, which confirmed the same results. Mauchly’s test was used to assess sphericity, and when violated, Greenhouse–Geisser corrections were applied. Effect sizes were reported using partial eta squared (η^2^), with values of 0.01, 0.06, and 0.14 interpreted as small, medium, and large effects, respectively [30]. When a significant main effect or interaction involving Time was observed, Bonferroni-adjusted post hoc pairwise comparisons were performed to examine differences between time points. Effect sizes for all non-significant Time × Supplement interactions were consistently small (η^2^ < 0.05), indicating that the absence of significant findings was not attributable to insufficient statistical power to detect medium or large effects. Baseline values were compared between intervention periods and did not differ between supplement conditions for any outcome, indicating no evidence of carryover effects. Statistical significance was set at *p* < 0.05.

## 3. Results

### 3.1. Maximal Voluntary Torque

**Figure 2:**
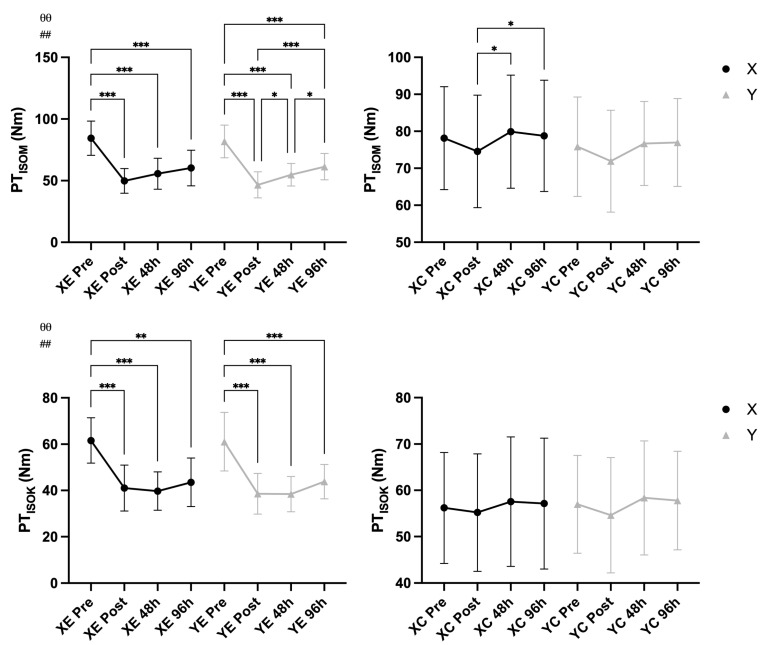
Peak isometric (PT_ISOM_) and isokinetic (PT_ISOK_) voluntary torque (mean ± SD) across time points in supplement (X) vs. placebo (Y) conditions and exercised (E) vs. control (C) arms. Notes: * *p* < 0.05, ** *p* < 0.01, *** *p* < 0.001 for pairwise comparisons across time (Bonferroni-adjusted); θθ *p* < 0.01 for main effect of time; ## *p* < 0.01 for Time × EIMD interaction.

A significant main effect of time was observed, indicating an overall change in peak torque (F = 4.77, *p* = 0.006, η^2^ = 0.078) and isokinetic torque (F = 7.62, *p* < 0.001, η^2^ = 0.120) across time points. A significant Time × EIMD interaction revealed that peak (F = 5.88, *p* = 0.002, η^2^ = 0.095) and isokinetic (F = 15.46, *p* < 0.001, η^2^ = 0.216) torque changes differed between the exercised and control arms. No significant interaction was found for Time × Supplement or Time × EIMD × Supplement (*p* > 0.05, η^2^ < 0.105), suggesting that the supplementation did not significantly influence torque loss-recovery dynamics (Figure 2).

### 3.2. Arm Swelling and Range of Motion

**Figure 3:**
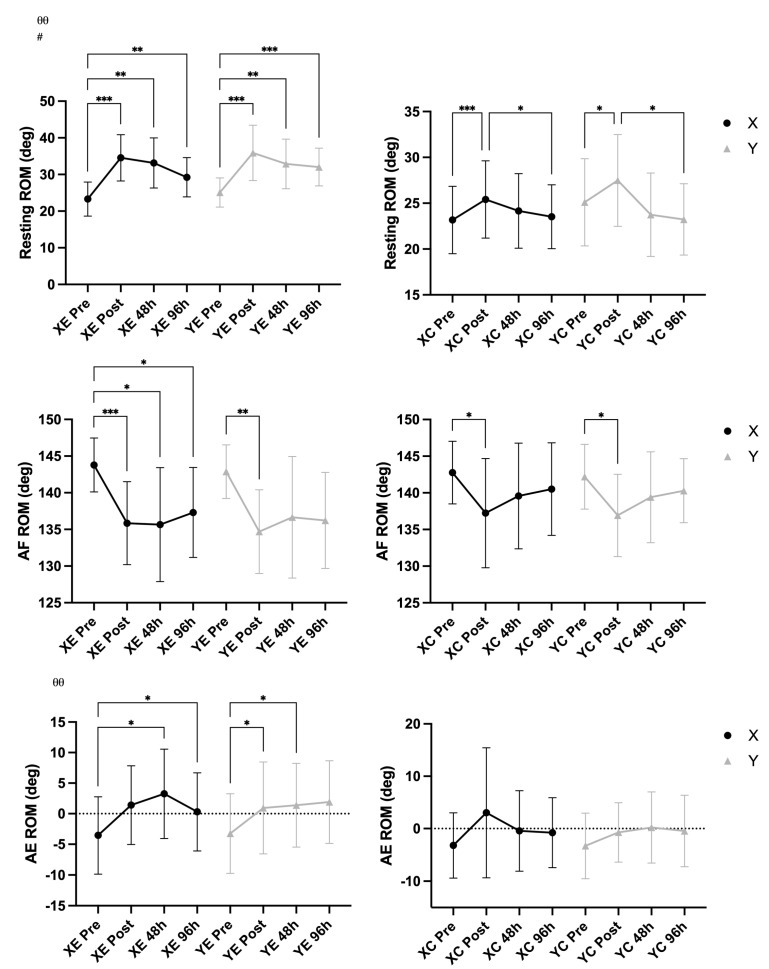
Range of motion (ROM) at rest, in arm flexion (AF), and arm extension (AE) (mean ± SD), across time points in supplement (X) vs. placebo (Y) conditions and exercised (E) vs. control (C) arms. Notes: * *p* < 0.05, ** *p* < 0.01, *** *p* < 0.001 for pairwise comparisons across time (Bonferroni-adjusted); θθ *p* < 0.01 for main effect of time; # *p* < 0.05 for Time × EIMD interaction.

**Figure 4:**
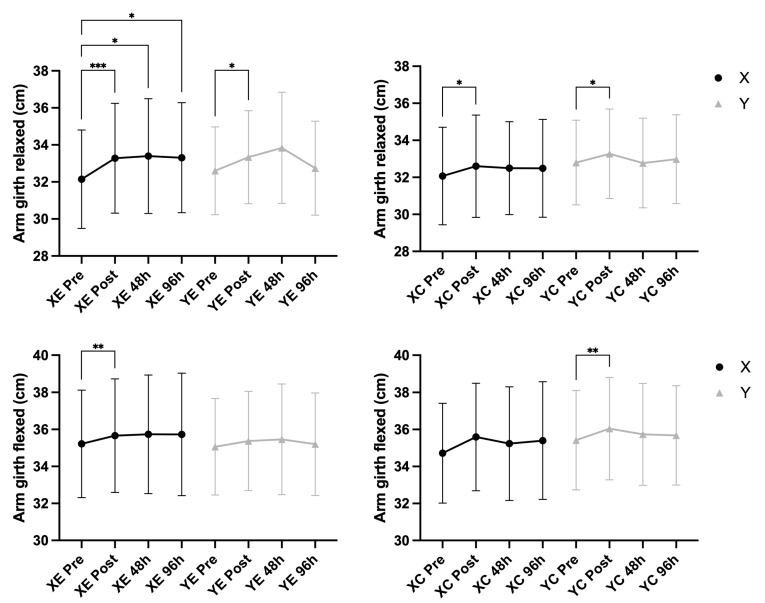
Arm girth measured in relaxed and flexed positions (mean ± SD) across time points in supplement (X) vs. placebo (Y) conditions and exercised (E) vs. control (C) arms. Notes: * *p* < 0.05, ** *p* < 0.01, *** *p* < 0.001 for pairwise comparisons across time (Bonferroni-adjusted); No significant main or interaction effects were observed (all *p* > 0.05).

A significant main effect of time was observed for ROM at rest (F = 14.02, *p* < 0.001, η^2^ = 0.200) and in the extended position (F = 17.00, *p* < 0.001, η^2^ = 0.233), indicating overall changes across time points. A significant Time × EIMD interaction was found for ROM at rest (F = 3.86, *p* = 0.017, η^2^ = 0.064), showing that changes differed between the exercised and control arms. No significant main effects or interactions were observed for ROM in the flexed position (*p* > 0.05). Arm girth, measured in both relaxed and flexed conditions, did not show any significant changes over time or interactions with EIMD or supplement (all *p* > 0.05), suggesting minimal or no swelling response detectable at the gross anatomical level. The supplementation condition did not significantly influence any ROM or arm girth outcomes (Figure 3 and Figure 4).

### 3.3. Muscle and Tendon Swelling

**Figure 5:**
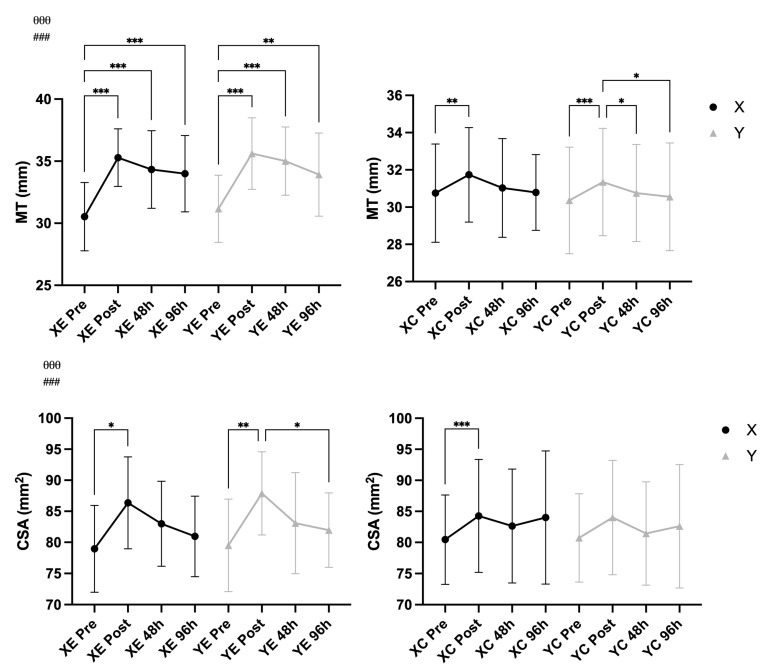
Muscle thickness (MT) and cross-sectional area (CSA) (mean ± SD) across time points in supplement (X) vs. placebo (Y) conditions and exercised (E) vs. control (C) arms. Notes: * *p* < 0.05, ** *p* < 0.01, *** *p* < 0.001 for pairwise comparisons across time (Bonferroni-adjusted); θθθ *p* < 0.001 for main effect of time; ### *p* < 0.001 for Time × EIMD interaction.

**Figure 6:**
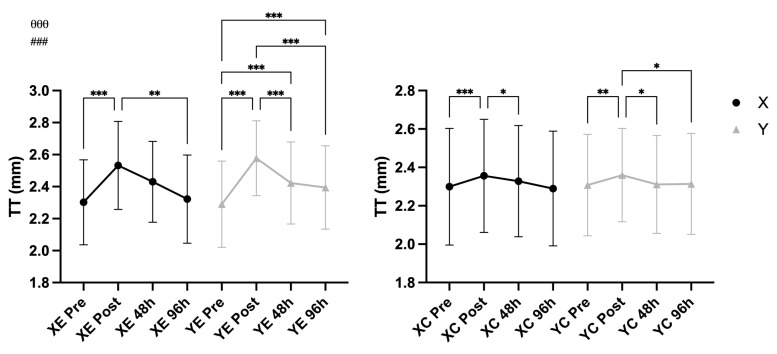
Tendon thickness (TT) (mean ± SD) across time points in supplement (X) vs. placebo (Y) conditions and exercised (E) vs. control (C) arms. Notes: * *p* < 0.05, ** *p* < 0.01, *** *p* < 0.001 for pairwise comparisons across time (Bonferroni-adjusted); θθθ *p* < 0.001 for main effect of time; ### *p* < 0.001 for Time × EIMD interaction.

A significant main effect of time was observed for MT (F = 76.34, *p* < 0.001, η^2^ = 0.577), CSA (F = 28.80, *p* < 0.001, η^2^ = 0.340), and TT (F = 61.68, *p* < 0.001, η^2^ = 0.524), indicating clear structural changes over time. A significant Time × EIMD interaction was also found for each variable: MT (F = 39.39, *p* < 0.001, η^2^ = 0.413), CSA (F = 6.59, *p* < 0.001, η^2^ = 0.105), and TT (F = 24.83, *p* < 0.001, η^2^ = 0.307), suggesting that these changes differed between the exercised and control arms. No significant Time × Supplement or Time × EIMD × Supplement interactions were observed for any of the three markers (all *p* > 0.05), indicating that the supplementation condition did not influence muscle or tendon morphological responses to eccentric exercise (Figure 5 and Figure 6).

### 3.4. Echo Intensity

**Figure 7:**
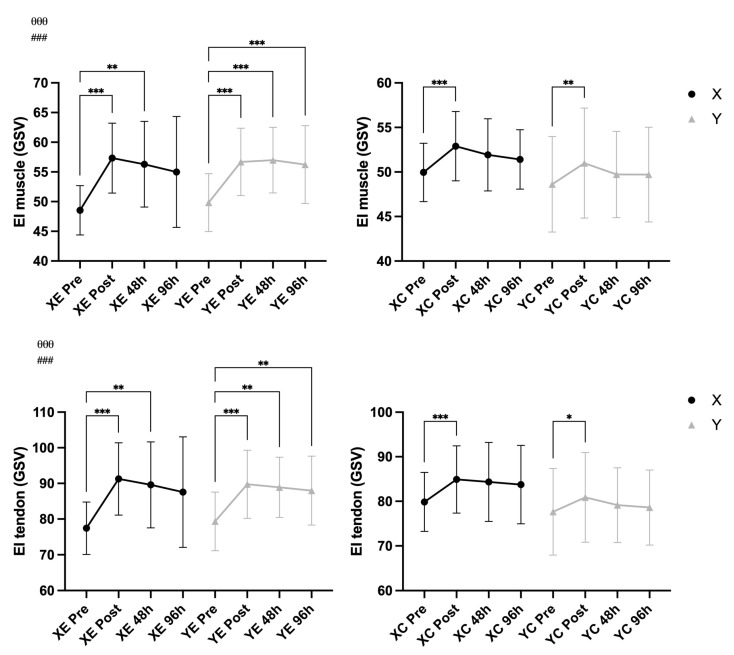
Biceps brachii muscle and tendon echo intensity (EI) (mean ± SD) across time points in supplement (X) vs. placebo (Y) conditions and exercised (E) vs. control (C) arms. Notes: * *p* < 0.05, ** *p* < 0.01, *** *p* < 0.001 for pairwise comparisons across time (Bonferroni-adjusted); θθθ *p* < 0.001 for main effect of time; ### *p* < 0.001 for Time × EIMD interaction.

A significant main effect of time was observed for biceps brachii muscle EI (F = 38.17, *p* < 0.001, η^2^ = 0.405) and tendon EI (F = 35.09, *p* < 0.001, η^2^ = 0.397). A significant Time × EIMD interaction (muscle EI: F = 10.00, *p* < 0.001, η^2^ = 0.151; tendon EI: F = 9.17, *p* < 0.001, η^2^ = 0.139) revealed that EI changes differed between the exercised and control arms. No significant effects were found for Time × Supplement or Time × EIMD × Supplement, suggesting that the supplementation did not influence the echo intensity response (Figure 7).

### 3.5. CK and Muscle Soreness

**Figure 8:**
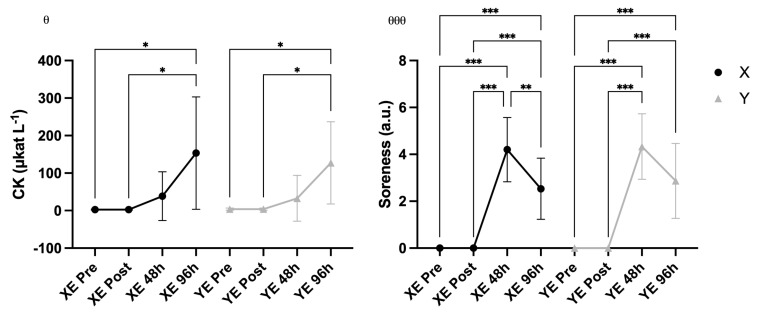
Creatine kinase (CK) and muscle soreness (mean ± SD) across time points in supplement (X) vs. placebo (Y) conditions for the exercised arm (E). Notes: * *p* < 0.05, ** *p* < 0.01, *** *p* < 0.001 for pairwise comparisons across time (Bonferroni-adjusted); θ *p* < 0.05 for main effect of time, θθθ *p* < 0.001 for main effect of time.

A significant main effect of time was observed for CK concentration (F = 4.58, *p* = 0.023, η^2^ = 0.141), indicating changes in circulating muscle damage markers over time following eccentric exercise. However, no significant Time × Supplement interaction was found (*p* = 0 0.411) (Figure 7). For muscle soreness, a significant main effect of time was observed (F = 151.94, *p* < 0.001, η^2^ = 0.884). No significant Time × Supplement interaction was detected (*p* = 0.649) (Figure 8). Supplementary Friedman tests of muscle soreness confirmed the same pattern of results.

## 4. Discussion

The present study investigated the potential modulatory effect of acute broccoli-derived glucoraphanin supplementation on the physiological responses to eccentric EIMD. As expected, the eccentric protocol led to significant impairments across multiple domains, including range of motion, muscle strength, structural morphology, and perceived soreness, confirming substantial muscle damage. All markers were gradually returning to baseline during the monitored recovery period of 96 h. We hypothesized that short-term glucoraphanin supplementation would attenuate indices of EIMD and accelerate recovery of muscle–tendon morphology and voluntary torque. This hypothesis was not supported: no Time × Supplement interactions emerged for any outcome.

Functional markers, such as ROM and both isometric and isokinetic voluntary torque, were significantly reduced following EIMD, with gradual recovery observed over time. These impairments were specific to the exercised arm, as evidenced by significant Time × EIMD interactions. However, no modulatory effect of glucoraphanin supplementation was detected. While the supplement was hypothesized to preserve contractile function by attenuating oxidative damage and inflammation-induced neuromuscular inhibition [13,15,17], our data did not support this hypothesis. These findings suggest that acute glucoraphanin supplementation does not produce measurable benefits for muscle function recovery in the days following eccentric exercise.

Although a previous short-term study has linked glucoraphanin with a reduction in oxidative stress and subjective muscle soreness [15], no such effects were evident in the present study. Plasma CK levels and muscle soreness ratings increased significantly, peaking later than structural markers, with similar trajectories in both the glucoraphanin and placebo groups. Additionally, increased thickness of the biceps brachii and its distal tendon was observed over time, consistent with acute eccentric exercise induction of transient inflammatory edema and structural remodeling [4,31]. Muscle swelling is typically attributed to localized fluid accumulation and leukocyte infiltration triggered by sarcomeric disruption and secondary inflammation [4,21]. In parallel, the acute increase in tendon thickness is therefore plausibly explained by localized inflammation and associated peritendinous fluid accumulation [32,33]. Although we hypothesized that glucoraphanin might attenuate this swelling [13,15,17], none of the measured markers supported this hypothesis.

Overall, while the eccentric protocol effectively elicited robust and localized muscle-tendon responses across structural, functional, and perceptual domains, acute intake of high-glucoraphanin broccoli powder did not influence the extent of these changes or the time course of recovery. This contrasts with findings of some other studies of sulforaphane, the bioactive compound derived from glucoraphanin, which have shown promise in having the potential to reduce functional impairments after intense exercise. For example, Ruhee et al. (2025) demonstrated that sulforaphane supplementation reduced oxidative stress and preserved muscle function in mice following exhaustive treadmill running [16], and Komine et al. (2021) reported accelerated recovery of muscle soreness and range of motion, along with reduced oxidative stress markers, after acute eccentric exercise bout following two weeks of sulforaphane intake (i.e., 30 mg or 3 tablets of 10 mg of glucoraphanin each per meal per day for 2-weeks) in a human pilot trial [15]. Notably, the supplementation strategy used by Komine et al. differed substantially from the present study in terms of glucoraphanin dose and cumulative exposure duration [15]. Their protocol involved a higher daily intake administered over more than two weeks prior to exercise. Such differences in exposure magnitude and duration may be relevant when interpreting divergent findings across studies, particularly given that sulforaphane-mediated effects are thought to involve transcriptional and redox-sensitive signalling mechanisms.

Furthermore, Flockhart et al. reported that a similar 7 days of glucosinolate-rich broccoli sprout intake during daily intense training lowered skeletal muscle protein carbonyls, reduced circulating myeloperoxidase, and improved exercise performance compared with placebo, indicating that benefits may emerge under repeated dosing plus cumulative training stress [34].

One possible explanation for the absence of an effect in our study is that the applied eccentric protocol may have been too demanding, particularly in untrained individuals, and potentially overwhelming the modest antioxidant or anti-inflammatory benefits achievable with short-term glucoraphanin intake. The magnitude of EIMD observed here may simply exceed the capacity of acute nutraceutical interventions to yield meaningful protective effects [35,36]. Because our eccentric bout was deliberately severe to ensure robust EIMD, it likely exceeded loads encountered in most training, rehabilitation, or daily contexts; therefore, generalizability is limited, and modest protective effects that might emerge under milder or repeated submaximal loading could have been masked. Future studies might consider lower-intensity or daily-life-like eccentric loading protocols to better detect subtle protective effects of dietary bioactives, especially in non-athletic or clinical populations. Moreover, sulforaphane’s reported benefits in chronic disease models may require sustained exposure or long-term supplementation protocols to manifest, potentially through epigenetic modulation of redox-sensitive genes [37]. The compound acts primarily through activation of the Nrf2 signaling pathway, which regulates the expression of antioxidant and cytoprotective genes [11,12,13]. This mechanism involves transcriptional and epigenetic processes, including histone acetylation and DNA methylation, and may require repeated or prolonged intake to exert biological effects [11,12,13,37,38]. Therefore, although Nrf2 activation can occur rapidly after sulforaphane exposure, sustained gene expression and cytoprotective enzyme induction often require repeated dosing over several days to weeks, which may explain why our 7-day protocol provided insufficient cumulative signaling [13,39].

These findings should also be interpreted within the context of our sample, which included generally healthy but non-athletic individuals. Trained athletes with higher adaptive reserves or repeated stress exposure might show different responsiveness to glucoraphanin supplementation. Conversely, non-athletic individuals may also exhibit heightened responsiveness to protective interventions due to lower baseline antioxidant capacity, an aspect that warrants direct investigation.

In parallel, we aimed to follow changes in muscle and tendon EI, a marker associated with fluid accumulation, connective tissue disruption, and cellular infiltration. Though EI is not a classical marker of EIMD, it responded similarly in terms of changes over time to structural markers, increasing significantly in the exercised arm only. Based on its sensitivity to edema and early-stage tissue remodeling after eccentric exercise, EI was expected to be modulated by broccoli supplementation due to its inherent anti-inflammatory effects. Sulforaphane, as a bioactive metabolite of glucoraphanin metabolism, has been shown to enhance phase II detoxification enzyme activity and suppress inflammatory cascades [13,15,17], which could potentially mitigate such structural alterations. Nevertheless, no effect of the broccoli supplement on EI was observed, which may reflect a suboptimal supplementation protocol relative to the severity of the induced muscle damage.

An additional finding relates to the timeline of changes in EI relative to classical structural, functional, and biochemical markers of EIMD. Muscle and tendon swelling, ROM loss, and strength deficits peaked immediately post-exercise and returned toward baseline by 48 h [27,40,41]. In contrast, plasma CK activity and muscle soreness followed a delayed trajectory, aligning with previous findings that dissociate mechanical from systemic markers of muscle damage [29]. CK release is influenced by membrane disruption and lymphatic transport, which may delay its peak, while soreness arises from complex nociceptive and inflammatory pathways [29]. Interestingly, EI showed its most pronounced elevation immediately after exercise, resembling the time course of morphological changes rather than systemic or perceptual markers. This immediate increase in EI likely reflects intramuscular fluid shifts and early-stage inflammation, as increased EI has been associated with edema, infiltration, and disorganized muscle structure on imaging [41]. Unlike plasma CK activity and muscle soreness, EI appears more sensitive to acute, localized responses in the muscle tissue, offering complementary insight into early-stage tissue disruption. The dissociation between EI and delayed markers reinforces the notion that no single metric can comprehensively capture the full extent of EIMD and highlights the value of employing a multimodal assessment approach when characterizing EIMD and evaluating the effects of potential recovery interventions. By integrating mechanical, structural, biochemical, and perceptual markers, researchers and practitioners can more accurately map the sequence of events following muscle-damaging exercise and better tailor recovery strategies to specific phases of tissue remodeling.

### Limitations and Perspectives

The broccoli powder was reconstituted in boiling water, which may have diminished plant myrosinase activity and thus limited immediate sulforaphane formation; this conversion may have been reliant on gut microbiota, which are known to exhibit considerable variability. Furthermore, sulforaphane metabolites, Nrf2 target engagement, and genotype/phenotype modifiers of isothiocyanate metabolism (e.g., GST polymorphisms) were neither controlled nor assessed, and actual exposure to antioxidant sulforaphane likely varied between participants to an unknown extent. These factors may have attenuated any supplement effects. Moreover, while the chosen dose and timing of glucoraphanin supplementation were modeled according to the existing literature, the acute timeframe may not have allowed for sufficient bioaccumulation or downstream transcriptional activation. Consequently, future studies should consider chronic supplementation models, potentially combined with gene expression profiling of target tissues or cells. Additionally, although ultrasound and biochemical markers were employed to monitor muscle and tendon responses, direct molecular indicators of Nrf2 activation or oxidative stress were not measured. It should also be noted that intrarater reliability for ultrasound measurements was established from repeat scans in only two participants. The blinding of outcomes was not examined, although both the broccoli and placebo were matched in appearance and preparation. The male-only sample limits generalizability, and the dietary intake beyond cruciferous vegetables was not comprehensively controlled. Although the crossover design reduced inter-individual variability and the results of the a priori post hoc sensitivity analysis, the limited sample size may have limited sensitivity to detect very small effects. The male-only sample limits generalizability to females, particularly as potential sex differences in sulforaphane metabolism or EIMD responsiveness remain largely unexplored. Finally, exercise protocols eliciting lower levels of muscle damage, which may be more pertinent to rehabilitation or daily activities, could reveal potential benefits of the compound. Accordingly, future research should investigate alternative glucoraphanin supplementation strategies, including extended intake durations and varied timing protocols, in conjunction with more ecologically valid exercise interventions. Such approaches may elucidate whether broccoli-derived bioactives support recovery under moderate physiological stress and contribute to musculoskeletal health in the general population. Although the sample size was modest, the crossover design helped mitigate inter-individual variability; nevertheless, the presence of very small supplement effects cannot be definitively excluded.

## 5. Conclusions

Acute high-dose supplementation with glucoraphanin-rich broccoli powder prepared with hot water did not significantly alter the extent of eccentric exercise-induced muscle damage or the rate of recovery across structural, functional, biochemical, and perceptual outcomes under the conditions tested, in the absence of confirmed sulforaphane bioavailability. To better understand glucoraphanin’s role in musculoskeletal resilience and recovery, future studies should examine longer supplementation periods with repeated daily dosing, employ less severe or repeated submaximal eccentric loading protocols, and consider preparations that preserve or standardize myrosinase activity to enhance sulforaphane availability. Importantly, these null findings provide critical evidence to refine intervention strategies and prevent misplaced expectations regarding the efficacy of acute glucoraphanin supplementation for recovery from severe eccentric muscle damage in untrained individuals.

## Data Availability

Raw data are available upon reasonable request to the corresponding author.

## Abbreviations

The following abbreviations are used in this manuscript:

**Abbreviation**	**Definition**
AF	Arm flexion
AE	Arm extension
ANOVA	Analysis of variance
B-mode	Brightness mode (ultrasound imaging)
CC BY	Creative Commons Attribution
CK	Creatine kinase
CSA	Cross-sectional area
CV	Coefficient of variation
DOMS	Delayed onset muscle soreness
E	Exercised arm
EI	Echo intensity
EIMD	Exercise-induced muscle damage
ICC	Intraclass correlation coefficient
MT	Muscle thickness
NIH	National Institutes of Health
NQO1	NAD(P)H quinone oxidoreductase 1
Nrf2	Nuclear factor erythroid 2–related factor 2
PTISOK	Peak isokinetic torque
PTISOM	Peak isometric torque
ROM	Range of motion
SD	Standard deviation
TT	Tendon thickness
Y	Placebo condition
X	Supplement condition
η^2^	Partial eta squared
